# Genome-Wide Identification and Expression Analysis of the NAC Gene Family in *Kandelia obovata*, a Typical Mangrove Plant

**DOI:** 10.3390/cimb44110381

**Published:** 2022-11-13

**Authors:** Man-Man Sun, Xiu Liu, Xiao-Juan Huang, Jing-Jun Yang, Pei-Ting Qin, Hao Zhou, Ming-Guo Jiang, Hong-Ze Liao

**Affiliations:** 1 Guangxi Key Laboratory of Polysaccharide Materials and Modification, School of Marine Sciences and Biotechnology, Guangxi Minzu University, 158 West Daxue Road, Nanning 530008, China; 2 Guangxi Key Laboratory of Special Non-Wood Forest Cultivation and Utilization, Guangxi Forestry Research Institute, 23 Yongwu Road, Nanning 530002, China

**Keywords:** abiotic stress, cold stress, *Kandelia obovata*, mangrove, NAC transcription factor

## Abstract

The NAC (*NAM*, *ATAF1/2*, and *CUC2*) gene family, one of the largest transcription factor families in plants, acts as positive or negative regulators in plant response and adaption to various environmental stresses, including cold stress. Multiple reports on the functional characterization of NAC genes in *Arabidopsis thaliana* and other plants are available. However, the function of the NAC genes in the typical woody mangrove (*Kandelia obovata*) remains poorly understood. Here, a comprehensive analysis of NAC genes in K. obovata was performed with a pluri-disciplinary approach including bioinformatic and molecular analyses. We retrieved a contracted NAC family with 68 genes from the *K. obovata* genome, which were unevenly distributed in the chromosomes and classified into ten classes. These *Ko*NAC genes were differentially and preferentially expressed in different organs, among which, twelve up-regulated and one down-regulated *Ko*NAC genes were identified. Several stress-related cis-regulatory elements, such as LTR (low-temperature response), STRE (stress response element), ABRE (abscisic acid response element), and WUN (wound-responsive element), were identified in the promoter regions of these 13 *Ko*NAC genes. The expression patterns of five selected *Ko*NAC genes (*Ko*NAC6, *Ko*NAC15, *Ko*NAC20, *Ko*NAC38, and *Ko*NAC51) were confirmed by qRT-PCR under cold treatment. These results strongly implied the putative important roles of *Ko*NAC genes in response to chilling and other stresses. Collectively, our findings provide valuable information for further investigations on the function of *Ko*NAC genes.

## 1. Introduction

Transcription factors (TFs) are of immense importance due to their crucial impact on controlling the transcription rate by binding to the cis-regulatory elements, resulting in activation or inhibition of the transcription level of target genes [1]. There are numerous types of TF families in plants, among which the NAC (*NAM*, *ATAF1/2*, and *CUC2*) family serves as one of the largest plant-specific TF families and is named after the *Petunia hybrida* E. Vilm. *NO APICAL MERISTEM* (NAM) [2] and *Arabidopsis thaliana* (L.) Heynh. genes *ATAF1/2* and *CUP-SHAPED COTYLEDON 2* (*CUC2*) [3]. A typical NAC protein contains an N-terminal conserved NAC domain for DNA binding and nuclear localization and a variable C-terminal region with transcriptional regulatory activity [4].

As a complex plant-specific family, the NAC genes with considerable quantities are present in a wide range of species. A large number of NAC TFs have been identified in various plants, including *A. thaliana* [5], *Actinidia eriantha* Benth. [6], *Asparagus officinalis* L. [7], *Betula pendula* Roth [8], *Hylocereus undatus* (Haw.) Britton & Rose [9], *Juglans mandshurica* Maxim. [10], *Medicago sativa* L. [11], *Miscanthus sinensis* Andersson [12], banana (*Musa acuminata* Colla) [13], *Oryza sativa* L. [5], *Populus trichocarpa* Torr. & A. Gray ex Hook. [14], *Salix psammophila* C. Wang & Chang Y. Yang [15], *Solanum lycopersicum* L. [16], *Zanthoxylum bungeanum* Maxim. [17], and *Zea mays* L. [18]. Multiple lines of evidence illustrate that NAC genes act as positive or negative regulators involved in diverse biological processes, including plant response and adaptation to cold and other abiotic stresses [19,20]. *Ma*NAC1, one banana NAC TF, acts as a downstream target of MaICE1 and interacts with the C-repeat binding factor *Ma*CBF1, conferring fruit cold tolerance [21]. Two overexpressed NAC genes from *H. undatus*, *Hu*NAC20 and *Hu*NAC25, confer enhanced cold tolerance of transgenic *A. thaliana* plants [9]. Overexpression of *Mb*NAC*25* from *Malus baccata* (L.) Borkh. improves the resistance against chilling stress through enhanced scavenging capability of reactive oxygen species (ROS) in transgenic *A. thaliana* plants [22]. The tomato NAC gene *NAM3* and its upstream regulator *miR164a* positively modulates cold tolerance by inducing ethylene synthesis in tomato plants [23]. *CaNAC035*, a novel NAC gene from *Capsicum annuum*, was shown to positively regulate cold stress in company with its upstream TF gene *Cab*HLH79 [24]. *Ca*NAC064, another NAC gene from *C. annuum*, is strongly induced by chilling stress and positively modulates cold stress tolerance via interacting with low temperature-induced haplo-proteinase proteins [25]. Additionally, NAC TFs also function as negative regulators in response to low temperature. Overexpression of *Md*NAC029, an apple NAC gene, reduces cold tolerance in apple and *A. thaliana* via a CBF-dependent pathway [26]. *Ma*NAC25 and *Ma*NAC28, two NAC genes from banana, negatively regulate cold tolerance in fruits by upregulating the expression levels of phospholipid degradation genes [27].

Mangroves are a dominant halophytic vegetation with significant ecological value in various tropical and subtropical coastal wetlands and are well-adapted to these highly stressful intertidal regions [28,29]. Among them, *Kandelia obovata* Sheue C.R., H.Y. Liu & J.W.H. Yong is regarded as a typical true mangrove due to its highest natural distribution latitude, indicating that *K. obovata* possesses stronger resistance against low temperature in contrast to other mangroves [30,31]. Various physiological evidences have shown that *K. obovata* displays better performance when exposed to chilling stress than other mangrove plants [32,33,34]. However, the underlying molecular mechanisms of cold response and adaptation in *K. obovata* are largely unknown. Here, we describe the genome-wide identification and expression analysis of *K. obovata* NAC (*Ko*NAC) genes in response to low temperature based on its available chromosome-level reference genome [35] with a pluri-disciplinary approach including bioinformatic and molecular analyses, hopefully providing valuable insights into the function of NAC genes in cold response and breeding for cold resistance.

## 2. Materials and Methods

### 2.1. Identification and Chromosomal Distribution of NAC TFs in K. obovata

The *K. obovata* chromosome-scale genome (2n = 2x= 36) was obtained from Genome Warehouse (https://bigd.big.ac.cn/gwh) (accessed on 8 March 2022) under accession number GWHACBH00000000 [35]. The Hidden Markov Model (HMM) file for NAM domain (PF02365) was downloaded from Pfam database (https://pfam.xfam.org/) [36] (accessed on 8 March 2022), and was used to retrieve the NAC proteins with a cut-off value of 0.001 by HMMER 3.3.2 (http://hmmer.org/download.html) [37] (accessed on 8 March 2022). BLASTP (basic local alignment search tool for proteins) against *K. obovata* genome data with *A. thaliana* NAC protein sequences (Table S1) retrieved from The *Arabidopsis* Information Resource (TAIR, https://www.arabidopsis.org/) [38] (accessed on 10 March 2022) was implemented (*e*-value = 0.001). Taking these two results together, the final members of the *Ko*NAC genes were acquired and verified by PfamScan (*e*-value = 0.001, https://www.ebi.ac.uk/Tools/pfa/pfamscan/) (accessed on 12 March 2022) [39] and NCBI’s conserved domain database (NCBI-CDD) (*e*-value = 0.001, https://www.ncbi.nlm.nih.gov/cdd/) (accessed on 12 March 2022) [40]. The basic information for *Ko*NAC gene, including chromosome localization, intron number, average intron length, protein length, and isoelectric point (*pI*) values was determined based on the genome database. The chromosomal distribution map of *Ko*NAC genes was drawn using MapChart 2.32 (https://www.wur.nl/en/show/Mapchart.htm) (accessed on 15 March 2022) [41].

### 2.2. Phylogenetic Analysis of NAC Proteins

The amino acid sequences of the NAC members of *K. obovata* and *A. thaliana* were aligned using Clustal X, and a neighbor-joining unrooted phylogenetic tree with 1000 bootstrap replications was constructed by MEGA 7.0 (www.megasoftware.net) (accessed on 15 March 2022) [42]. Finally, the tree was further modified by iTOL v6.5.8 (https://itol.embl.de/) (accessed on 15 March 2022) [43].

### 2.3. Gene Structure, Motif Identification, and Collinearity Analysis

The intron/exon structure of *Ko*NAC genes was determined with the online gene structure display server (http://gsds.gao-lab.org/) (accessed on 18 March 2022) [44]. The conserved motifs in *Ko*NAC proteins were identified by MEME suite v5.4.1 (http://meme-suite.org/) (accessed on 18 March 2022) [45]. The collinearity relationship of the *K. obovata* NAC genes between *A. thaliana* [5] and *P. trichocarpa* [14] were analyzed by MCScanX (http://chibba.pgml.uga.edu/mcscan2/) (accessed on 18 March 2022) [46]. These results were presented and visualized using TBtools (https://github.com/CJ-Chen/Tbtools) (accessed on 18 March 2022) [47].

### 2.4. Expression Analysis of KoNAC Genes Based on Public RNA-Seq Data

Two previously released RNA-seq data sets of *K. obovata* were introduced here to analyze the expression profiles of *Ko*NAC genes. The expression patterns of *Ko*NAC genes in eight organs (root, stem, leaf, flower, pistil, stamen, sepal, and fruit) were obtained according to the previously published transcriptomic data under the NCBI BioProject accession number PRJNA416402 (https://www.ncbi.nlm.nih.gov/bioproject) (accessed on 31 March 2022) [31]. The expression levels of *Ko*NAC genes in response to cold stress were determined based on the publicly released data from the NCBI BioProject under accession number PRJNA678025. These two RNA-seq data were remapped back to the *K. obovata* genome used here [35]. All expression data were normalized as fragments per kilobase of transcript per million fragments mapped (FPKM) values [48]. The differentially expressed genes (DEGs) related to chilling stress were defined under the criteria of fold change (FC) ≥ 1.5. The expression profiles of *Ko*NAC genes were visualized as heatmaps using TBtools [47].

### 2.5. Plant Materials and Treatment

The healthy mature propagules of the typical viviparous mangrove plant *K. obovata* were sampled from Guangxi Maoweihai Mangrove Nature Reserve, Qinzhou, China (21°37′23″ N, 108°44′13″ E) and cultured in the Mangrove Germplasm Resources Center (MGRC) of Guangxi Forestry Research Institute (GFRI) (Figure S1). The seedlings were grown in plastic pots containing sand and cultivated in a growth chamber at 28 °C and 75% humidity with a photoperiod of 14 h light/10 h darkness, and watered weekly with half-strength Hoagland’s nutrient solution [49]. At the eight-leaf stage, the seedlings were treated under low temperature (4 °C) for 0 h, 6 h, 12 h, and 24 h, respectively. All treatments were performed with three replicates. The leaves were harvested, immediately frozen in liquid nitrogen, and stored at −80 °C for RNA extraction.

### 2.6. Cis-Regulatory Element Analysis of the KoNAC Genes

The upstream 1500 bp promoter sequences from the ATG start codon of the *Ko*NAC genes were retrieved from the *K. obovata* genome, and the cis-regulatory elements in the promoter regions were predicted using Plant CARE (https://bioinformatics.psb.ugent.be/webtools/plantcare/html/) (accessed on 31 March 2022) [50] and displayed by TBtools [47].

### 2.7. Quantitative Real-Time PCR Assays

Total RNA was extracted from the sampled leaves mentioned above using TRIzol (Invitrogen, http://www.invitrogen.com) (accessed on 20 April 2022). Quantitative real-time PCR (qRT-PCR) assays were conducted, as described previously [51], using an ABI PRISM 7500 Real-time PCR System (Applied Biosystem) with 2^−ΔΔCT^ method [52]. The specific primers of *Ko*NAC genes used here are listed in Table S2. The actin gene (GWHTACBH010383.1) was used as an internal control. Student’s *t*-test in statistical analysis was performed using Graphpad Prism 9.0.0 (https://www.graphpad-prism.cn/) (accessed on 28 April 2022).

## 3. Results

### 3.1. Genome-Wide Identification of the K. obovata NAC Genes

**Figure 1:**
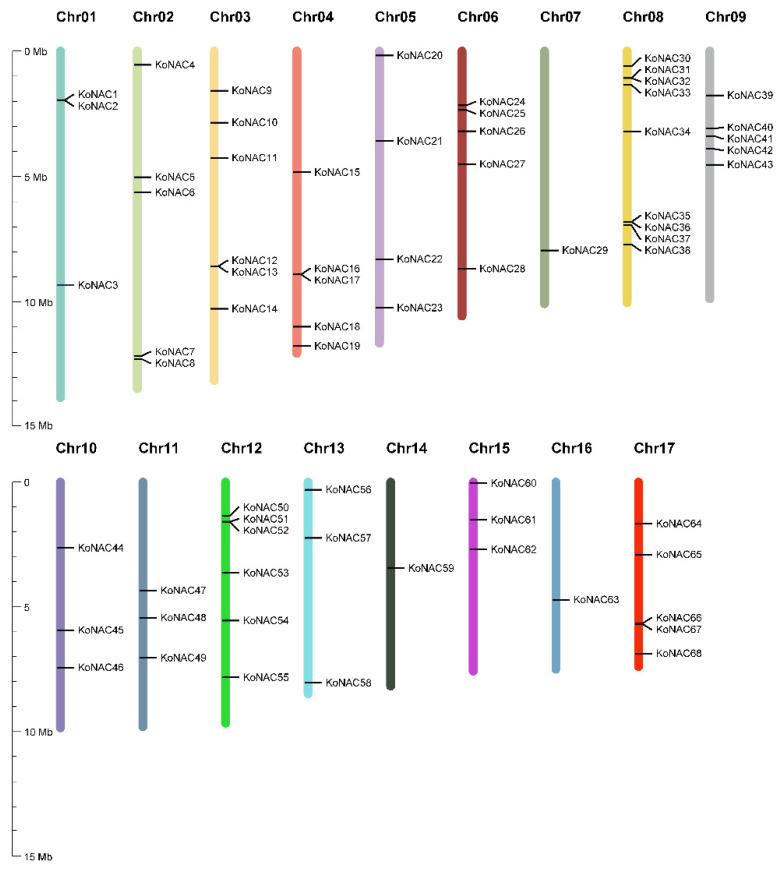
Distribution of *Ko*NAC genes in *K. obovata* genome. The 68 *Ko*NAC genes were unevenly distributed on 17 chromosomes (Chr01–Chr17) denoted in different colors, while no *Ko*NAC gene was found on Chr18. Values on the y-axis indicate the chromosome length and gene position.

Two independent strategies for retrieval of *Ko*NAC genes from the *K. obovata* genome, HMM search and BLASTP, were used here. Taken together, 68 putative *Ko*NAC genes were identified and confirmed by PfamScan and NCBI-CDD. Based on their chromosome location, these *Ko*NAC genes were named *Ko*NAC1 to *Ko*NAC68 and unevenly distributed on 17 chromosomes (Chrs), with no *Ko*NAC gene present on Chr18 (Table 1, Figure 1). Detail-wise, nine *Ko*NAC genes were located on Chr08, six genes were located on both Chr03 and Chr12, and five *Ko*NAC genes each were located on Chr02, Chr04, Chr06, Chr09, and Chr17, while only one *Ko*NAC gene each was found on Chr07, Chr14, and Chr16.

Moreover, every *Ko*NAC gene contained one or more introns with an average length of 371 bp, while the proteins encoded by *Ko*NAC genes ranged from 162 amino acid (aa) residues (*Ko*NAC61) to 638 aa (*Ko*NAC60) in length, with an average length of 344 aa. The pI values varied from 4.20 (*Ko*NAC45) to 10.07 (*Ko*NAC11), over half of the members (39/68) exhibiting *pI* > 7 (Table 1).

### 3.2. Phylogenetic Analysis and Classification of KoNAC Proteins

**Figure 2:**
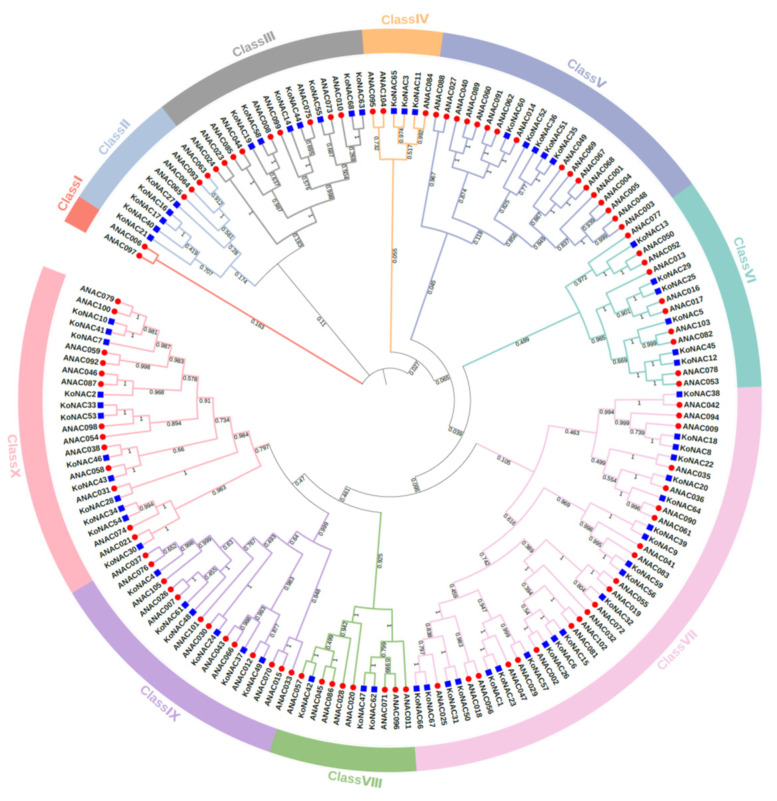
Phylogenetic analysis of the NAC proteins from *K. obovata* and *A. thaliana*. The unrooted tree was constructed by MEGA 7.0 based on the neighbor-joining (NJ) method with 1000 bootstrap replications. The NAC proteins of *K. obovata* (*Ko*NAC) in blue squares, and *A. thaliana* (*At*NAC) in red circles were classified into ten classes represented with different colors.

**Figure 3:**
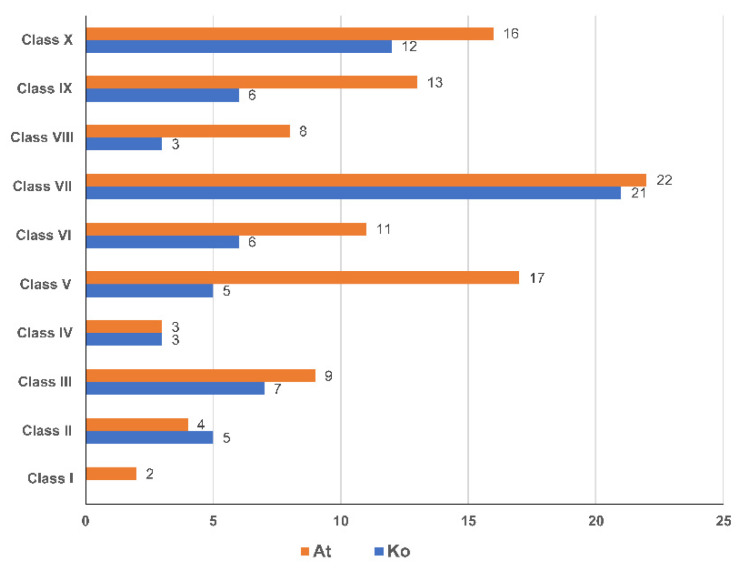
Classification comparison between *K. obovata* (*Ko*) and *A. thaliana* (*At*) NAC proteins.

To illustrate the phylogenetic relationship among *K. obovata* and *Arbidopsis thaliana* NAC proteins, a neighbor-joining phylogenetic tree was constructed with 68 *Ko*NAC proteins and 105 *At*NAC proteins (Table S1). The result showed that the 173 NAC proteins could be classified into ten classes, namely, Class I to Class X (Figure 2). Obviously, Class VII, with 21 *Ko*NACs and 24 *A*NACs was the largest class, followed by Class X with 12 *Ko*NACs and 14 *A*NACs. Other classes contained no more than 10 *Ko*NACs each. Specially, no *Ko*NAC belonged to Class I (Figure 3).

**Figure 4:**
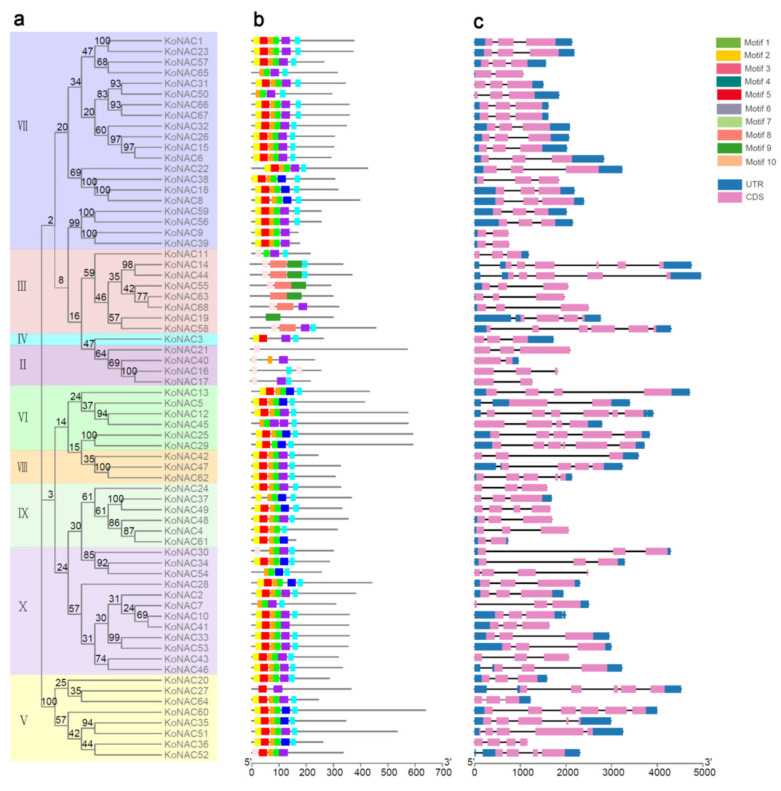
Gene structure and motif organization of the *Ko*NAC genes. (**a**) The unrooted NJ tree of *Ko*NAC proteins was constructed using MEGA 7.0, while different classes were represented in different colors. Bootstrap values from 1000 replicates are shown on the nodes. (**b**) Ten different conserved motifs of *Ko*NAC proteins were identified using MEME software. Different colored boxes indicate different motifs. (**c**) The structures of *Ko*NAC genes are shown, including UTR regions (blue box), exons (grey boxes), and introns (black lines). UTR, untranslated region; CDS, coding sequence of a gene.

To better understand the phylogenetic relationship and classification of *Ko*NAC genes, the gene structure and motif organization of the 68 *Ko*NAC genes were analyzed. Each *Ko*NAC gene had one or more introns and contained no more than six exons, while over half of the *Ko*NAC genes (41/68) contained three exons (Figure 4c). Additionally, a total of 10 conserved motifs were queried within all *K. obovata* NAC proteins. Most motifs were located within the N-terminal region (Figure 4b), and motif 1, motif 2, motif 4, and motif 5 were the common elements in *Ko*NAC genes. Clearly, these results showed that the *Ko*NAC genes in the same phylogenetic cluster harbored similar gene structures and motif compositions (Figure 4), which further supported the evolutionary relationship of *Ko*NAC genes demonstrated above.

### 3.3. Collinearity Analysis of KoNAC Genes

**Figure 5:**
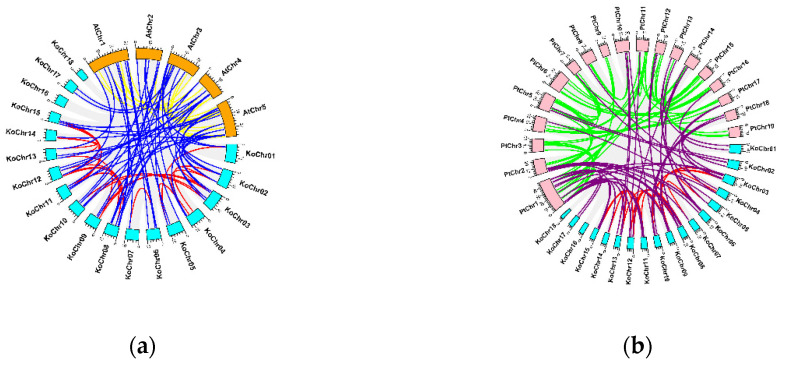
Collinearity analysis of *Ko*NAC genes. (**a**) Genes in 5 chromosomes of *A. thaliana* (*At*Chrs), in orange, and 18 chromosomes of *K. obovata* (*Ko*Chrs), in cyan, are introduced here. The orthologous pairs between *Ko*NACs and *A*NACs are highlighted in blue. The gene pairs among *A*NACs are colored in yellow, while the gene pairs among *KoNAC*s are colored in red. (**b**) Genes in 19 chromosomes of *P. trichocarpa* (*Pt*Chrs), in pink, and 18 chromosomes of *K. obovata* (*Ko*Chrs), in cyan, are introduced here. The orthologous pairs between *Ko*NACs and *Pt*NACs are highlighted in purple. The gene pairs among *Pt*NACs are colored in green, while the gene pairs among *Ko*NACs are colored in red.

It is well-known that *P. trichocarpa* is a typical model plant for functional genomics and molecular studies in woody species. Moreover, *K. obovata* (Rhizophoraceae) and *P. trichocarpa* (Salicaceae) belong to the same order, Malpighiales (https://www.ncbi.nlm.nih.gov/Taxonomy/Browser/wwwtax.cgi) (accessed on 18 March 2022). Therefore, to better investigate the evolutionary relationship of NAC genes, the collinearity analysis was performed based on the genomes of *A. thaliana* and *P. trichocarpa* (Figure 5). There were 16,355 collinear gene pairs between *K. obovata* and *A. thaliana* identified, among which 52 orthologous gene pairs between *Ko*NACs and *At*NACs were obtained (Figure 5a, blue lines). Meanwhile, a total of 26,594 collinear gene pairs between *K. obovata* and *P. trichocarpa* were available, among which 54 orthologous gene pairs between *KoNAC*s and *Pt*NACs were determined (Figure 5b, purple lines). Taken together, 49 common *Ko*NAC genes shared homologous relationships with both *A. thaliana* and *P. trichocarpa* NAC genes (Table S3), implying these genes might function in a similar manner. In the meantime, there were 11 non-orthologous *Ko*NAC genes (*Ko*NAC13, *Ko*NAC16, *Ko*NAC17, *Ko*NAC21, *Ko*NAC40, *Ko*NAC50, *Ko*NAC53*, Ko*NAC63, *Ko*NAC65, *Ko*NAC67, and *Ko*NAC68) compared to NAC genes of *A. thaliana* and *P. trichocarpa*. These genes displayed different structure (Figure 4), among which four genes (*Ko*NAC16, *Ko*NAC17, *Ko*NAC21, *Ko*NAC40) clustered as the subgroup of Class II (Figure 2).

### 3.4. Expression Patterns of KoNAC Genes in Different Organs

**Figure 6:**
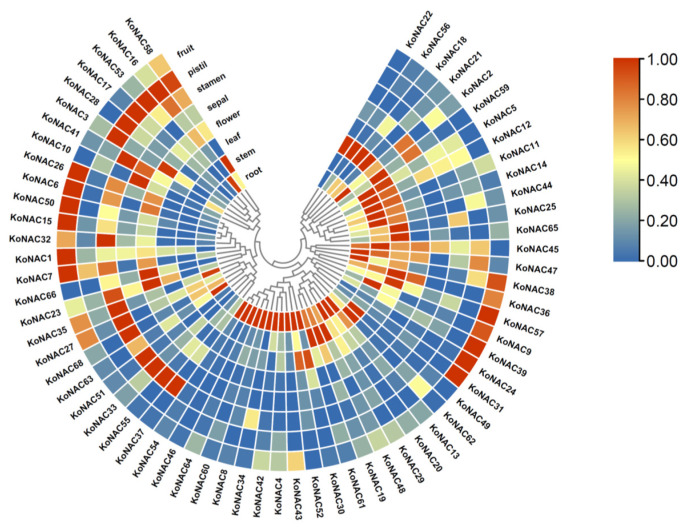
Expression patterns of *Ko*NAC genes in different organs. The transcript levels of the *Ko*NAC genes in eight organs of *K. obovata* were determined based on published transcriptomic data (NCBI BioProject: PRJNA416402). The color scale indicates increasing expression levels from blue to red. Deeper red colors represent higher expression levels, while darker blue colors indicate lower values.

To gain an insight into the function of NAC genes in *K. obovata*, the expression levels of all *Ko*NAC genes in various organs, including root, stem, leaf, flower, pistil, stamen, sepal, and fruit were determined based on previously published RNA-seq data of *K. obovata*. Noticeably, the expression patterns of *Ko*NAC genes were not in a constitutive mode, whereas they were differentially and preferentially expressed in different organs (Figure 6, Table S4). For example, 28 out of 68 *Ko*NAC genes were highly expressed in roots, while 18 *Ko*NAC genes were preferentially expressed in leaves, and 13 *Ko*NAC genes were mainly expressed in fruits.

### 3.5. Expression Analysis of KoNAC Genes under Cold Treatment

**Figure 7:**
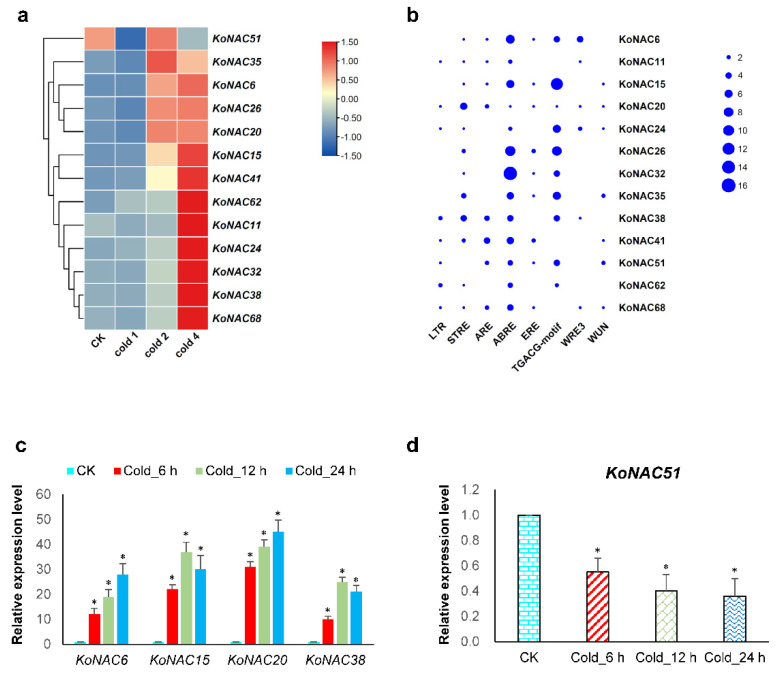
Expression analysis of *Ko*NAC genes under cold treatment. (**a**) The transcript levels of the *Ko*NAC genes in response to cold were determined based on publicly available RNA-seq data (NCBI BioProject: PRJNA678025). Deeper red colors represent higher expression levels of up-regulated *Ko*NAC genes, while darker blue colors indicate higher values of down-regulated *Ko*NAC genes. Cold 1, first-time cold treatment; Cold 2, second-time cold treatment; Cold 4, fourth-time cold treatment. (**b**) The cis-regulatory elements in the promoters of the 13 *Ko*NAC genes were predicated by PlantCARE. Eight well-known stress-related elements were identified. The size of the blue ball indicates the number of the elements in the *Ko*NAC promoters. Expression levels of four upregulated *Ko*NAC genes (**c**) and one down-regulated *Ko*NAC (**d**) under cold treatment were confirmed by qRT-PCR. Three independent experiments were performed. The actin gene in *K. obovata* acted as the internal control. Asterisks indicate significant differences compared with CK by Student’s *t*-test. *, *p* < 0.05.

To gain more insight into the function of *Ko*NAC genes, the expression profiles of these genes under cold treatment were detected based on the public transcriptomic data of *K. obovata*. There were 13 *Ko*NAC genes differentially expressed in response to chilling stress, among which one down-regulated *Ko*NAC gene (*Ko*NAC51) and 12 up-regulated *Ko*NACs (*Ko*NAC6, *Ko*NAC11, *Ko*NAC15, *Ko*NAC20, *Ko*NAC24, *Ko*NAC26, *Ko*NAC32, *Ko*NAC35, *Ko*NAC38, *Ko*NAC41, *Ko*NAC62, and *Ko*NAC68) were available (Figure 7a, Table S5). Specifically, four different genes, *Ko*NAC6, *Ko*NAC15, *Ko*NAC20, and *Ko*NAC38, were largely upregulated with higher and more significant values after treatment. The expression levels of these four up-regulated and one down-regulated *Ko*NACs were confirmed by qRT-PCR (Figure 7c,d), implying that these *Ko*NAC genes might act as positive or negative regulators in response to chilling stress.

### 3.6. Stress-Related Cis-Regulatory Elements Identified in KoNAC Genes

To obtain more evidence for the differentially expressed *Ko*NAC genes on stress responses, the cis-regulatory elements in the promoter regions of these 13 *Ko*NAC genes were predicated. Consequently, 8 well-known stress-related elements were available (Figure 7b, Table S6). LTR (low-temperature response; CCGAAA), a core cis-acting element involved in cold stress response, was present in the majority of the detected *Ko*NAC genes. STRE (stress response element; AGGGG) and ARE (antioxidant response element; AAACCA) were two types of regulatory elements in rapid response to anaerobic stress and environmental stimuli. ABRE (abscisic acid response element; ACGTG), ERE (ethylene response element; ATTTTAAA), and TGACG-motifs were responsible for stress induction by three major stress-related hormones, ABA, ethylene, and methyl jasmonate (MeJA), respectively. Additionally, two biotic stress-responsive elements, WRE3 (wound-response element 3; CCACCT) and WUN-motif (wound-responsive element; AAATTACT) were found in several detected promoters, as well.

## 4. Discussion

The NAC gene family, one of the largest TF families in plants, was used as positive or negative regulators in response to environmental stimuli including cold stress [19]. Multiple investigations on the functional characterization of NAC genes were reported for *A. thaliana* [5,53,54], *P. trichocarpa* [14], and other plants [19]. However, the function of the NAC genes in the typical woody mangrove *K. obovata* responding to abiotic stresses remains largely unknown. Here, we identified a contracted NAC gene family with 68 members from the *K. obovata* genome. These *Ko*NAC genes were differentially and preferentially expressed in various organs, and 13 *Ko*NAC genes were differentially expressed under cold treatment based on the publicly available RNA-seq data.

*Ko*NAC proteins, in company with *A. thaliana* NAC proteins were categorized into 10 classes according to phylogenetic analysis. Obviously, the *K. obovata* NAC family exhibited a significant contraction in number compared to the NAC families in *A. thaliana* [5], *P. trichocarpa* [14], and other plants [15,16], and the decreased *Ko*NAC genes in class V, class VIII, and class IX mainly contributed to the contraction (Figure 3). These results are consistent with the previous findings [35], and the contraction might relate to the evolutionary adaption to the intertidal zones. Moreover, 49 genes from the contracted *Ko*NAC family shared orthologous relationships with the NAC genes of *A. thaliana* and *P. trichocarpa*, implying these genes might have similar functions [55]. Additionally, compared to *At*NAC and *Ko*NAC genes, there existed 11 non-orthologous *Ko*NAC genes, among which, four genes (*Ko*NAC16, *Ko*NAC17, *Ko*NAC21, and *Ko*NAC40) clustered in class II (Figure 2). Another non-orthologous gene, *Ko*NAC68, was induced under cold treatment, implying it might potentially function in response to cold stress in *K. obovata* (Figure 7a). More attention should be paid to these non-orthologous genes, and functional investigations of these genes will provide valuable knowledge about mangrove species.

To explore the function of NAC genes in *K. obovata*, the expression patterns of all *KoNAC*s in various organs were determined. In contrast to the constitutive expression patterns of other gene families [56,57,58], *KoNAC* genes were differentially and preferentially expressed in different organs. For instance, there were 28 *Ko*NAC genes expressed highly in roots, 18 *Ko*NAC genes expressed preferentially in leaves, and 13 *Ko*NAC genes expressed mainly in fruits. Referentially, the root-expressed gene *Os*NAC2 modulated root development in rice by involving the crosstalk of auxin and cytokinin pathways [59]. The *A. thaliana* rosette-expressed gene, *ANAC087*, positively regulated rosette development and leaf senescence [60]. *FaRIF*, a strawberry NAC gene, was reported as one key regulator controlling fruit ripening [61]. This evidence implied that these organ-specific *Ko*NAC genes might function as key regulators in organ development. Moreover, the organ expression patterns of NAC genes in *K. obovata* were not similar to that in *A. thaliana* and other plants. For instance, *Ko*NAC46 and *Ko*NAC54 were primarily expressed in roots (Figure 6), however, *A*NAC048 and *A*NAC074, the closest orthologues of these two *Ko*NAC genes (Figure 2), respectively, were expressed in different organs. *A*NAC048 was involved in vascular development [62], and *A*NAC074 positively regulated programmed cell death of stigmatic tissue in *A. thaliana* [63]. Therefore, functional characterization of the *Ko*NAC genes primarily expressed in roots distinct from other plants should be deeply covered in the future.

To better understand the roles of *Ko*NAC genes, the expression analysis under chilling stress was performed based on the public transcriptomic data. In total, 13 out of 68 *Ko*NAC genes were differentially expressed under cold treatment. Among them, *Ko*NAC51 was the only down-regulated gene, whereas its closest homologue *Ko*NAC35 was up-regulated after treatment, implying these two class V genes might function oppositely in response to cold stress. Half of the up-regulated genes (*Ko*NAC6, *Ko*NAC15, *Ko*NAC20, *Ko*NAC26, *Ko*NAC32, and *Ko*NAC38) belonged to the class VII subgroup (Figure 2 and Figure 7a). Particularly, *Ko*NAC6, *Ko*NAC15, and *Ko*NAC26 clustered together and shared high sequence similarity to their closest orthologs *A*NAC002, *A*NAC081, and *A*NAC102 in *A. thaliana*. *A*NAC002 (*ATAF1*) was reported to serve as dual regulators responsive to abiotic and biotic stresses [64,65,66]. *A*NAC081 (*ATAF2*) was rapidly induced by pathogen attack and involved in plant defense [67,68], while *A*NAC102 was responsive to low-oxygen and high-light stresses [69,70]. Overexpression of *Ml*NAC5, another closest ortholog of *Ko*NAC26 and *A*NAC002 from *Miscanthus lutarioriparius* L.Liu ex S.L.Chen & Renvoize, led to enhanced tolerance to cold and drought stresses in *A. thaliana* [71]. Additionally, three closest orthologs of *Ko*NAC32, *A*NAC019, *A*NAC055 and *A*NAC072, were required for drought tolerance in *A. thaliana* [72], among which *A*NAC019 and *A*NAC055 displayed a dual function in regulating ABA response and jasmonate response [73,74]. *A. thaliana A*NAC042, the closest ortholog of *Ko*NAC38, conferred stress tolerance through regulating phytohormone metabolism and signaling [75,76,77]. Moreover, various stress-related cis-regulatory elements were identified from the promoters of these *Ko*NAC genes (Figure 7b). The LTR element is an indispensable cis-acting element in plant response to low temperature [78,79]. Deletion of the LTR element will result in complete loss of promoter activity under cold stress [79]. STRE is a common cis-regulatory element in eukaryotes, and involved in response to multiple environmental stimuli [80]. ARE is an antioxidant response element in rapid response to anaerobic stress [81]. Meanwhile, ABRE, ERE, and TGACG motifs are three major types of elements related to plant hormones (ABA, ethylene, and MeJA) [82,83,84]. Among them, ABRE and TGACG motifs are enriched in the majority of the detected *Ko*NAC genes, implying these *Ko*NAC genes might respond to stresses via hormone-mediated pathways [85]. Additionally, both WRE3 and WUN motifs are biotic stress-responsive elements and present in several detected promoters as well, implying the *Ko*NAC genes might function in response to biotic stresses [86,87]. Taken together, these findings suggest that these *Ko*NAC genes may be involved responses to other abiotic or biotic stresses in addition to the cold response, providing auxiliary evidence for these *Ko*NAC genes in response to abiotic and biotic stresses. To know more about the function of *Ko*NAC genes, further investigation and more proof are required.

## 5. Conclusions

In the present study, a pluri-disciplinary work concerning comprehensive analysis of the *Ko*NAC gene family was performed. We identified a contracted NAC TF family containing 68 genes from the genome of the typical mangrove plant *K. obovata* based on bioinformatic analysis. These *Ko*NAC genes were unevenly distributed in 17 chromosomes of *K. obovata*. The NAC genes of *K. obovata* and *A. thaliana* were classified into ten classes, while no *Ko*NAC gene belonged to class I. Obviously, the decreased members of class V, class VIII, and class IX mainly contributed to the contraction of the *Ko*NAC family. *Ko*NAC genes were differentially and preferentially expressed in different organs. Among them, 13 *Ko*NAC genes were rapidly induced by chilling stress. The expression patterns of five selected *Ko*NAC genes (*Ko*NAC6, *Ko*NAC15, *Ko*NAC20, *Ko*NAC38, and *Ko*NAC51) were confirmed by qRT-PCR. Additionally, several stress-related cis-acting elements were detected in the promoter regions of these *Ko*NAC genes, implying *Ko*NAC genes might participate in multiple stress responses. Summarily, our findings will provide positive references for further investigations on functional characterization of *Ko*NAC genes in stress responses.

## Figures and Tables

**Table 1:** Basic information of *K. obovata* NAC genes.

Name	Gene ID	Class	Chromosome Position	Intron Number	Average Intron Length (bp)	Protein Length (aa)	*pI*
*KoNAC1*	GWHPACBH000260.1	VII	Chr01: 1952420-1954549	2	210	375	8.33
*KoNAC2*	GWHPACBH000261.1	X	Chr01: 1966355-1968293	2	213	381	6.38
*KoNAC3*	GWHPACBH001011.1	IV	Chr01: 9324213-9325936	2	179	268	9.76
*KoNAC4*	GWHPACBH001737.1	IX	Chr02: 545874-547928	2	540	314	4.72
*KoNAC5*	GWHPACBH002133.1	VI	Chr02: 5018997-5022392	2	627	414	4.56
*KoNAC6*	GWHPACBH002150.1	VII	Chr02: 5630019-5632845	2	557	291	6.26
*KoNAC7*	GWHPACBH002927.1	X	Chr02: 12147292-12149791	2	695	308	9.72
*KoNAC8*	GWHPACBH002942.1	VII	Chr02: 12271496-12273886	2	265	397	6.91
*KoNAC9*	GWHPACBH003351.1	VII	Chr03: 1575855-1576587	1	152	170	10.01
*KoNAC10*	GWHPACBH003542.1	X	Chr03: 2845984-2847971	3	82	358	8.29
*KoNAC11*	GWHPACBH003714.1	IV	Chr03: 4257070-4258248	2	188	215	10.07
*KoNAC12*	GWHPACBH004035.1	VI	Chr03: 8571932-8575841	7	267	573	4.35
*KoNAC13*	GWHPACBH004037.1	VI	Chr03: 8580877-8585584	3	922	432	5.87
*KoNAC14*	GWHPACBH004257.1	III	Chr03: 10268576-10273325	6	484	340	8.05
*KoNAC15*	GWHPACBH005193.1	VII	Chr04: 4833704-4835719	2	237	301	6.63
*KoNAC16*	GWHPACBH005487.1	II	Chr04: 8899908-8901721	2	518	259	7.96
*KoNAC17*	GWHPACBH005488.1	II	Chr04: 8902766-8904022	1	590	220	8.48
*KoNAC18*	GWHPACBH005795.1	VII	Chr04: 10981103-10983284	2	229	317	9.64
*KoNAC19*	GWHPACBH005903.1	III	Chr04: 11744358-11747114	4	148	304	5.55
*KoNAC20*	GWHPACBH005980.1	VII	Chr05: 171989-173574	2	182	285	8.57
*KoNAC21*	GWHPACBH006496.1	II	Chr05: 3585985-3588070	2	179	576	5.03
*KoNAC22*	GWHPACBH006945.1	VII	Chr05: 8286321-8289550	2	626	425	7.89
*KoNAC23*	GWHPACBH007161.1	VII	Chr05: 10223818-10225996	2	171	372	8.40
*KoNAC24*	GWHPACBH007671.1	IX	Chr06: 2167096-2168677	2	300	327	6.13
*KoNAC25*	GWHPACBH007697.1	VI	Chr06: 2347486-2351319	4	385	591	4.47
*KoNAC26*	GWHPACBH007806.1	VII	Chr06: 3191353-3193417	2	295	303	6.78
*KoNAC27*	GWHPACBH007934.1	II	Chr06: 4507683-4512207	5	551	365	5.29
*KoNAC28*	GWHPACBH008265.1	X	Chr06: 8671993-8674297	2	374	441	6.35
*KoNAC29*	GWHPACBH009271.1	VI	Chr07: 7943036-7946752	5	272	592	4.37
*KoNAC30*	GWHPACBH009626.1	X	Chr08: 595220-599512	2	1624	300	7.03
*KoNAC31*	GWHPACBH009686.1	VII	Chr08: 1067480-1068977	2	114	343	9.60
*KoNAC32*	GWHPACBH009687.1	VII	Chr08: 1074862-1076943	2	171	347	8.70
*KoNAC33*	GWHPACBH009711.1	X	Chr08: 1347371-1350316	2	636	357	7.67
*KoNAC34*	GWHPACBH009970.1	X	Chr08: 3208565-3211847	2	1096	286	7.10
*KoNAC35*	GWHPACBH010230.1	V	Chr08: 6804416-6807401	4	274	345	5.82
*KoNAC36*	GWHPACBH010231.1	V	Chr08: 6808948-6810099	3	123	261	4.47
*KoNAC37*	GWHPACBH010248.1	IX	Chr08: 6936960-6938647	2	191	366	7.37
*KoNAC38*	GWHPACBH010352.1	VII	Chr08: 7704131-7705968	2	435	305	6.78
*KoNAC39*	GWHPACBH010982.1	VII	Chr09: 1762983-1763731	1	160	176	9.98
*KoNAC40*	GWHPACBH011181.1	II	Chr09: 3089069-3090025	1	112	236	6.50
*KoNAC41*	GWHPACBH011224.1	X	Chr09: 3378802-3380434	2	115	356	7.92
*KoNAC42*	GWHPACBH011287.1	VIII	Chr09: 3895422-3899009	2	1261	243	4.62
*KoNAC43*	GWHPACBH011356.1	X	Chr09: 4526977-4529037	2	553	318	7.21
*KoNAC44*	GWHPACBH012132.1	III	Chr10: 2632918-2637871	5	584	373	7.08
*KoNAC45*	GWHPACBH012473.1	VI	Chr10: 5929512-5932298	3	255	574	4.20
*KoNAC46*	GWHPACBH012540.1	X	Chr10: 7425092-7428318	3	579	333	9.19
*KoNAC47*	GWHPACBH012930.1	VIII	Chr11: 4340404-4343638	4	342	326	5.37
*KoNAC48*	GWHPACBH013040.1	IX	Chr11: 5432389-5434082	2	285	354	6.19
*KoNAC49*	GWHPACBH013264.1	IX	Chr11: 7036403-7038056	3	165	331	6.75
*KoNAC50*	GWHPACBH013622.1	VII	Chr12: 1361271-1363117	2	215	294	9.80
*KoNAC51*	GWHPACBH013650.1	V	Chr12: 1584267-1587516	4	219	534	4.43
*KoNAC52*	GWHPACBH013651.1	V	Chr12: 1588784-1591086	4	165	336	6.58
*KoNAC53*	GWHPACBH013779.1	X	Chr12: 3635952-3638945	2	591	354	8.79
*KoNAC54*	GWHPACBH013961.1	X	Chr12: 5551002-5553482	3	571	256	9.26
*KoNAC55*	GWHPACBH014293.1	III	Chr12: 7828847-7830888	2	494	296	8.61
*KoNAC56*	GWHPACBH014606.1	VII	Chr13: 304056-306202	2	230	255	9.29
*KoNAC57*	GWHPACBH014849.1	VII	Chr13: 2243283-2244839	2	109	265	7.90
*KoNAC58*	GWHPACBH015137.1	III	Chr13: 8039942-8044242	5	466	461	4.62
*KoNAC59*	GWHPACBH015463.1	VII	Chr14: 3448051-3450062	2	222	255	9.64
*KoNAC60*	GWHPACBH015782.1	V	Chr15: 43374-47367	5	324	638	4.57
*KoNAC61*	GWHPACBH015958.1	IX	Chr15: 1512975-1513703	1	125	162	9.55
*KoNAC62*	GWHPACBH016068.1	VIII	Chr15: 2698343-2700471	4	261	306	9.33
*KoNAC63*	GWHPACBH016810.1	III	Chr16: 4714504-4716464	2	516	304	9.25
*KoNAC64*	GWHPACBH017115.1	VII	Chr17: 1665396-1666616	2	119	245	8.31
*KoNAC65*	GWHPACBH017251.1	IV	Chr17: 2901510-2902567	1	103	314	7.77
*KoNAC66*	GWHPACBH017590.1	VII	Chr17: 5655463-5657073	2	147	358	8.88
*KoNAC67*	GWHPACBH017594.1	VII	Chr17: 5688319-5689928	2	146	358	8.88
*KoNAC68*	GWHPACBH017666.1	III	Chr17: 6866174-6868665	2	727	325	7.67

## Data Availability

The chromosome-scale genome of *Kandelia obovata* was obtained from Genome Warehouse under accession number GWHACBH00000000 (https://bigd.big.ac.cn/gwh) (accessed on 8 March 2022). The *Arabidopsis thaliana* genome was obtained from TAIR database (http://www.arabidopsis.org/) (accessed on 10 March 2022). The *Populus trichocarpa* genome version 2.0 was downloaded from Phytozome (http://www.phytozome.net/poplar) (accessed on 18 March 2022). The RNA-seq data in eight different organs of *K. obovata* was available from NCBI BioProject under accession number PRJNA416402 (https://www.ncbi.nlm.nih.gov/bioproject) (accessed on 31 March 2022). The RNA-seq data related to cold treatment in *K. obovata* was obtained from NCBI BioProject under accession number PRJNA678025.

## Supplementary Materials

The following supporting information can be downloaded at: https://www.mdpi.com/article/10.3390/cimb44110381/s1, Figure S1: Morphological features of *K. obovata*; Table S1: The basic information of *A. thaliana* NAC genes; Table S2: The primers of *Ko*NAC genes in this study; Table S3: Common orthologous gene pairs of *K. obovata* between *A. thaliana* and *P. trichocarpa*; Table S4: The FPKM values of *Ko*NAC genes expressed in different organs; Table S5: The FPKM values of differentially expressed *Ko*NAC genes under cold treatment; Table S6: Number of stress-related cis-regulatory elements in the promoter regions of the differentially expressed *Ko*NAC genes.

## References

[1] Singh K., Foley R.C., Onate-Sanchez L. (2002). Transcription factors in plant defense and stress responses. Curr. Opin. Plant Biol..

[2] Souer E., van Houwelingen A., Kloos D., Mol J., Koes R. (1996). The *No Apical Meristem* gene of *Petunia* is required for pattern formation in embryos and flowers and is expressed at meristem and primordia boundaries. Cell.

[3] Aida M., Ishida T., Fukaki H., Fujisawa H., Tasaka M. (1997). Genes involved in organ separation in *Arabidopsis*: An analysis of the *Cup-Shaped cotyledon* mutant. Plant Cell.

[4] Olsen A.N., Ernst H.A., Leggio L.L., Skriver K. (2005). NAC transcription factors: Structurally distinct, functionally diverse. Trends Plant Sci..

[5] Ooka H., Satoh K., Doi K., Nagata T., Otomo Y., Murakami K., Matsubara K., Osato N., Kawai J., Carninci P. (2003). Comprehensive analysis of NAC family genes in *Oryza sativa* and *Arabidopsis thaliana*. DNA Res..

[6] Jia D., Jiang Z., Fu H., Chen L., Liao G., He Y., Huang C., Xu X. (2021). Genome-wide identification and comprehensive analysis of NAC family genes involved in fruit development in kiwifruit (*Actinidia*). BMC Plant Biol..

[7] Li C., Zhang J., Zhang Q., Dong A., Wu Q., Zhu X., Zhu X. (2022). Genome-wide identification and analysis of the NAC transcription factor gene family in garden Asparagus (*Asparagus officinalis*). Genes.

[8] Chen S., Lin X., Zhang D., Li Q., Zhao X., Chen S. (2019). Genome-wide analysis of nac gene family in *Betula pendula*. Forests.

[9] Hu X., Xie F., Liang W., Liang Y., Zhang Z., Zhao J., Hu G., Qin Y. (2022). *HuNAC20* and *HuNAC25*, two novel NAC genes from pitaya, confer cold tolerance in transgenic *Arabidopsis*. Int. J. Mol. Sci..

[10] Li X., Cai K., Pei X., Li Y., Hu Y., Meng F., Song X., Tigabu M., Ding C., Zhao X. (2021). Genome-wide identification of NAC transcription factor family in *Juglans mandshurica* and their expression analysis during the fruit development and ripening. Int. J. Mol. Sci..

[11] He F., Zhang L., Zhao G., Kang J., Long R., Li M., Yang Q., Chen L. (2022). Genome-wide identification and expression analysis of the NAC gene family in Alfalfa revealed its potential roles in response to multiple abiotic stresses. Int. J. Mol. Sci..

[12] Nie G., Yang Z., He J., Liu A., Chen J., Wang S., Wang X., Feng G., Li D., Peng Y. (2021). Genome-wide investigation of the NAC transcription factor family in *Miscanthus sinensis* and expression analysis under various abiotic stress. Front. Plant Sci..

[13] Li B., Fan R., Yang Q., Hu C., Sheng O., Deng G., Dong T., Li C., Peng X., Bi F. (2020). Genome-wide identification and characterization of the NAC transcription factor family in *Musa Acuminata* and expression analysis during fruit ripening. Int. J. Mol. Sci..

[14] Hu R., Qi G., Kong Y., Kong D., Gao Q., Zhou G. (2010). Comprehensive analysis of NAC domain transcription factor gene family in *Populus trichocarpa*. BMC Plant Biol..

[15] Yang H., Fan L., Yu X., Zhang X., Hao P., Wei D., Zhang G. (2022). Analysis of the NAC gene family in *Salix* and the identification of *SpsNAC005* gene contributing to salt and drought tolerance. Forests.

[16] Jin J.F., Wang Z.Q., He Q.Y., Wang J.Y., Li P.F., Xu J.M., Zheng S.J., Fan W., Yang J.L. (2020). Genome-wide identification and expression analysis of the NAC transcription factor family in tomato (*Solanum lycopersicum*) during aluminum stress. BMC Genom..

[17] Hu H., Ma L., Chen X., Fei X., He B., Luo Y., Liu Y., Wei A. (2022). Genome-wide identification of the NAC gene family in *Zanthoxylum bungeanum* and their transcriptional responses to drought stress. Int. J. Mol. Sci..

[18] Peng X., Zhao Y., Li X., Wu M., Chai W., Sheng L., Wang Y., Dong Q., Jiang H., Cheng B. (2015). Genome-wide identification, classification and analysis of NAC type gene family in maize. J. Genet..

[19] Diao P., Chen C., Zhang Y., Meng Q., Lv W., Ma N. (2020). The role of NAC transcription factor in plant cold response. Plant Signal. Behav..

[20] Puranik S., Sahu P.P., Srivastava P.S., Prasad M. (2012). NAC proteins: Regulation and role in stress tolerance. Trends Plant Sci..

[21] Shan W., Kuang J.F., Lu W.J., Chen J.Y. (2014). Banana fruit NAC transcription factor MaNAC1 is a direct target of MaICE1 and involved in cold stress through interacting with MaCBF1. Plant Cell Environ..

[22] Han D., Du M., Zhou Z., Wang S., Li T., Han J., Xu T., Yang G. (2020). Overexpression of a *Malus baccata* NAC transcription factor gene *MbNAC25* increases cold and salinity tolerance in *Arabidopsis*. Int. J. Mol. Sci..

[23] Dong Y., Tang M., Huang Z., Song J., Xu J., Ahammed G.J., Yu J., Zhou Y. (2022). The miR164a-NAM3 module confers cold tolerance by inducing ethylene production in tomato. Plant J..

[24] Wang Z., Zhang Y., Hu H., Chen L., Zhang H., Chen R. (2022). *CabHLH79* acts upstream of *CaNAC035* to regulate cold stress in pepper. Int. J. Mol. Sci..

[25] Hou X.M., Zhang H.F., Liu S.Y., Wang X.K., Zhang Y.M., Meng Y.C., Luo D., Chen R.G. (2020). The NAC transcription factor *CaNAC064* is a regulator of cold stress tolerance in peppers. Plant Sci..

[26] An J.P., Li R., Qu F.J., You C.X., Wang X.F., Hao Y.J. (2018). An apple NAC transcription factor negatively regulates cold tolerance via CBF-dependent pathway. J. Plant Physiol..

[27] Song C., Wu M., Zhou Y., Gong Z., Yu W., Zhang Y., Yang Z. (2022). NAC-mediated membrane lipid remodeling negatively regulates fruit cold tolerance. Hortic. Res..

[28] Krauss K.W., McKee K.L., Lovelock C.E., Cahoon D.R., Saintilan N., Reef R., Chen L. (2014). How mangrove forests adjust to rising sea level. New Phytol..

[29] Nizam A., Meera S.P., Kumar A. (2022). Genetic and molecular mechanisms underlying mangrove adaptations to intertidal environments. iScience.

[30] Guo Z., Ma D., Li J., Wei M., Zhang L., Zhou L., Zhou X., He S., Wang L., Shen Y. (2022). Genome-wide identification and characterization of aquaporins in mangrove plant *Kandelia obovata* and its role in response to the intertidal environment. Plant Cell Environ..

[31] Su W., Ye C., Zhang Y., Hao S., Li Q.Q. (2019). Identification of putative key genes for coastal environments and cold adaptation in mangrove *Kandelia obovata* through transcriptome analysis. Sci. Total Environ..

[32] Chen L., Wang W., Li Q.Q., Zhang Y., Yang S., Osland M.J., Huang J., Peng C. (2017). Mangrove species’ responses to winter air temperature extremes in China. Ecosphere.

[33] Peng Y.-L., Wang Y.-S., Fei J., Sun C.-C., Cheng H. (2015). Ecophysiological differences between three mangrove seedlings (*Kandelia obovata*, *Aegiceras corniculatum*, and *Avicennia marina*) exposed to chilling stress. Ecotoxicology.

[34] Wang S.-M., Wang Y.-S., Su B.-Y., Zhou Y.-Y., Chang L.-F., Ma X.-Y., Li X.-M. (2022). Ecophysiological responses of five mangrove species (*Bruguiera gymnorrhiza*, *Rhizophora stylosa*, *Aegiceras corniculatum*, *Avicennia marina*, and *Kandelia obovata*) to chilling stress. Front. Mar. Sci..

[35] Hu M.J., Sun W.H., Tsai W.C., Xiang S., Lai X.K., Chen D.Q., Liu X.D., Wang Y.F., Le Y.X., Chen S.M. (2020). Chromosome-scale assembly of the *Kandelia obovata* genome. Hortic. Res..

[36] Mistry J., Chuguransky S., Williams L., Qureshi M., Salazar G.A., Sonnhammer E.L.L., Tosatto S.C.E., Paladin L., Raj S., Richardson L.J. (2021). Pfam: The protein families database in 2021. Nucleic Acids Res..

[37] Waack S., Keller O., Asper R., Brodag T., Damm C., Fricke W.F., Surovcik K., Meinicke P., Merkl R. (2006). Score-based prediction of genomic islands in prokaryotic genomes using hidden Markov models. BMC Bioinform..

[38] Lamesch P., Berardini T.Z., Li D., Swarbreck D., Wilks C., Sasidharan R., Muller R., Dreher K., Alexander D.L., Garcia-Hernandez M. (2012). The Arabidopsis Information Resource (TAIR): Improved gene annotation and new tools. Nucleic Acids Res..

[39] Madeira F., Park Y.M., Lee J., Buso N., Gur T., Madhusoodanan N., Basutkar P., Tivey A.R.N., Potter S.C., Finn R.D. (2019). The EMBL-EBI search and sequence analysis tools APIs in 2019. Nucleic Acids Res..

[40] Lu S., Wang J., Chitsaz F., Derbyshire M.K., Geer R.C., Gonzales N.R., Gwadz M., Hurwitz D.I., Marchler G.H., Song J.S. (2020). CDD/SPARCLE: The conserved domain database in 2020. Nucleic Acids Res..

[41] Voorrips R.E. (2002). MapChart: Software for the graphical presentation of linkage maps and QTLs. J. Hered..

[42] Kumar S., Stecher G., Tamura K. (2016). MEGA7: Molecular evolutionary genetics analysis version 7.0 for bigger datasets. Mol. Biol. Evol..

[43] Letunic I., Bork P. (2021). Interactive Tree Of Life (iTOL) v5: An online tool for phylogenetic tree display and annotation. Nucleic Acids Res..

[44] Hu B., Jin J., Guo A.Y., Zhang H., Luo J., Gao G. (2015). GSDS 2.0: An upgraded gene feature visualization server. Bioinformatics.

[45] Bailey T.L., Boden M., Buske F.A., Frith M., Grant C.E., Clementi L., Ren J., Li W.W., Noble W.S. (2009). MEME SUITE: Tools for motif discovery and searching. Nucleic Acids Res..

[46] Wang Y., Tang H., Debarry J.D., Tan X., Li J., Wang X., Lee T.H., Jin H., Marler B., Guo H. (2012). MCScanX: A toolkit for detection and evolutionary analysis of gene synteny and collinearity. Nucleic Acids Res..

[47] Chen C., Chen H., Zhang Y., Thomas H.R., Frank M.H., He Y., Xia R. (2020). TBtools: An integrative toolkit developed for interactive analyses of big biological data. Mol. Plant..

[48] Trapnell C., Williams B.A., Pertea G., Mortazavi A., Kwan G., van Baren M.J., Salzberg S.L., Wold B.J., Pachter L. (2010). Transcript assembly and quantification by RNA-Seq reveals unannotated transcripts and isoform switching during cell differentiation. Nat. Biotechnol..

[49] Du Z., You S., Zhao X., Xiong L., Li J. (2022). Genome-wide identification of WRKY genes and their responses to chilling stress in *Kandelia obovata*. Front. Genet..

[50] Lescot M., Dehais P., Thijs G., Marchal K., Moreau Y., Van de Peer Y., Rouze P., Rombauts S. (2002). PlantCARE, a database of plant cis-acting regulatory elements and a portal to tools for in silico analysis of promoter sequences. Nucleic Acids Res..

[51] Liao H.Z., Zhu M.M., Cui H.H., Du X.Y., Tang Y., Chen L.Q., Ye D., Zhang X.Q. (2016). *MARIS* plays important roles in *Arabidopsis* pollen tube and root hair growth. J. Integr. Plant Biol..

[52] Livak K.J., Schmittgen T.D. (2001). Analysis of relative gene expression data using real-time quantitative PCR and the 2^−ΔΔCT^ method. Methods.

[53] Guan Q., Yue X., Zeng H., Zhu J. (2014). The protein phosphatase RCF2 and its interacting partner NAC019 are critical for heat stress-responsive gene regulation and thermotolerance in *Arabidopsis*. Plant Cell.

[54] Yoo S.Y., Kim Y., Kim S.Y., Lee J.S., Ahn J.H. (2007). Control of flowering time and cold response by a NAC-domain protein in *Arabidopsis*. PLoS ONE.

[55] Gabaldon T., Koonin E.V. (2013). Functional and evolutionary implications of gene orthology. Nat. Rev. Genet..

[56] Han N., Tang R., Chen X., Xu Z., Ren Z., Wang L. (2021). Genome-wide identification and characterization of *WOX* genes in *Cucumis sativus*. Genome.

[57] Rahman H., Xu Y.P., Zhang X.R., Cai X.Z. (2016). *Brassica napus* genome possesses extraordinary high number of CAMTA genes and *CAMTA3* contributes to PAMP triggered immunity and resistance to *Sclerotinia sclerotiorum*. Front. Plant Sci..

[58] Yi H., Sardesai N., Fujinuma T., Chan C.W., Veena, Gelvin, S (2006). B. Constitutive expression exposes functional redundancy between the *Arabidopsis* histone H2A gene *HTA1* and other H2A gene family members. Plant Cell.

[59] Mao C., He J., Liu L., Deng Q., Yao X., Liu C., Qiao Y., Li P., Ming F. (2020). *OsNAC2* integrates auxin and cytokinin pathways to modulate rice root development. Plant Biotechnol. J..

[60] Vargas-Hernandez B.Y., Nunez-Munoz L., Calderon-Perez B., Xoconostle-Cazares B., Ruiz-Medrano R. (2022). The NAC transcription factor *ANAC087* induces aerial rosette development and leaf senescence in *Arabidopsis*. Front. Plant Sci..

[61] Martín-Pizarro C., Vallarino J.G., Osorio S., Meco V., Urrutia M., Pillet J., Casañal A., Merchante C., Amaya I., Willmitzer L. (2021). The NAC transcription factor *FaRIF* controls fruit ripening in strawberry. Plant Cell.

[62] Yang J.H., Lee K.-H., Du Q., Yang S., Yuan B., Qi L., Wang H. (2020). A membrane-associated NAC domain transcription factor XVP interacts with TDIF co-receptor and regulates vascular meristem activity. New Phytol..

[63] Gao Z., Daneva A., Salanenka Y., Van Durme M., Huysmans M., Lin Z., De Winter F., Vanneste S., Karimi M., Van de Velde J. (2018). KIRA1 and ORESARA1 terminate flower receptivity by promoting cell death in the stigma of *Arabidopsis*. Nat. Plants.

[64] Alshareef N.O., Otterbach S.L., Allu A.D., Woo Y.H., de Werk T., Kamranfar I., Mueller-Roeber B., Tester M., Balazadeh S., Schmockel S.M. (2022). NAC transcription factors ATAF1 and ANAC055 affect the heat stress response in *Arabidopsis*. Sci. Rep..

[65] Liu Y., Sun J., Wu Y. (2016). *Arabidopsis ATAF1* enhances the tolerance to salt stress and ABA in transgenic rice. J. Plant Res..

[66] Wu Y., Deng Z., Lai J., Zhang Y., Yang C., Yin B., Zhao Q., Zhang L., Li Y., Yang C. (2009). Dual function of *Arabidopsis ATAF1* in abiotic and biotic stress responses. Cell Res..

[67] Delessert C., Kazan K., Wilson I.W., Van Der Straeten D., Manners J., Dennis E.S., Dolferus R. (2005). The transcription factor ATAF2 represses the expression of pathogenesis-related genes in *Arabidopsis*. Plant J..

[68] Wang X., Culver J.N. (2012). DNA binding specificity of ATAF2, a NAC domain transcription factor targeted for degradation by Tobacco mosaic virus. BMC Plant Biol..

[69] Christianson J.A., Wilson I.W., Llewellyn D.J., Dennis E.S. (2009). The low-oxygen-induced NAC domain transcription factor ANAC102 affects viability of *Arabidopsis* seeds following low-oxygen treatment. Plant Physiol..

[70] D’Alessandro S., Ksas B., Havaux M. (2018). Decoding β-cyclocitral-mediated retrograde signaling reveals the role of a detoxification response in plant tolerance to photooxidative stress. Plant Cell.

[71] Yang X., Wang X., Ji L., Yi Z., Fu C., Ran J., Hu R., Zhou G. (2015). Overexpression of a *Miscanthus lutarioriparius* NAC gene *MlNAC5* confers enhanced drought and cold tolerance in *Arabidopsis*. Plant Cell Rep..

[72] Tran L.S.P., Nakashima K., Sakuma Y., Simpson S.D., Fujita Y., Maruyama K., Fujita M., Seki M., Shinozaki K., Yamaguchi-Shinozaki K. (2004). Isolation and functional analysis of *Arabidopsis* stress-inducible NAC transcription factors that bind to a drought-responsive cis-element in the early responsive to dehydration stress 1 promoter. Plant Cell.

[73] Bu Q., Jiang H., Li C.B., Zhai Q., Zhang J., Wu X., Sun J., Xie Q., Li C. (2008). Role of the *Arabidopsis thaliana* NAC transcription factors ANAC019 and ANAC055 in regulating jasmonic acid-signaled defense responses. Cell Res..

[74] Jiang H., Li H., Bu Q., Li C. (2009). The RHA2a-interacting proteins ANAC019 and ANAC055 may play a dual role in regulating ABA response and jasmonate response. Plant Signal. Behav..

[75] Ebrahimian-Motlagh S., Ribone P.A., Thirumalaikumar V.P., Allu A.D., Chan R.L., Mueller-Roeber B., Balazadeh S. (2017). JUNGBRUNNEN1 confers drought tolerance downstream of the HD-Zip I transcription factor AtHB13. Front. Plant Sci..

[76] Sakuraba Y., Bulbul S., Piao W., Choi G., Paek N.C. (2017). *Arabidopsis EARLY FLOWERING3* increases salt tolerance by suppressing salt stress response pathways. Plant J..

[77] Shahnejat-Bushehri S., Tarkowska D., Sakuraba Y., Balazadeh S. (2016). *Arabidopsis* NAC transcription factor JUB1 regulates GA/BR metabolism and signalling. Nat. Plants.

[78] Qian W., Xiao B., Wang L., Hao X., Yue C., Cao H., Wang Y., Li N., Yu Y., Zeng J. (2018). *CsINV5*, a tea vacuolar invertase gene enhances cold tolerance in transgenic *Arabidopsis*. BMC Plant Biol..

[79] Wu C., Zheng C., Ji G., Jiang P. (2019). Synergistic effects of HSE and LTR elements from hsp70 gene promoter of *Ulva prolifera* (Ulvophyceae, Chlorophyta) upon temperature induction. J. Phycol..

[80] Ebeed H.T. (2022). Genome-wide analysis of polyamine biosynthesis genes in wheat reveals gene expression specificity and involvement of STRE and MYB-elements in regulating polyamines under drought. BMC Genom..

[81] Fan S., Liu A., Zhang Z., Zou X., Jiang X., Huang J., Fan L., Zhang Z., Deng X., Ge Q. (2019). Genome-wide identification and expression analysis of the metacaspase gene family in *Gossypium* species. Genes.

[82] Watanabe K.A., Homayouni A., Gu L., Huang K.Y., Ho T.D., Shen Q.J. (2017). Transcriptomic analysis of rice aleurone cells identified a novel abscisic acid response element. Plant Cell Environ..

[83] Itzhaki H., Maxson J.M., Woodson W.R. (1994). An ethylene-responsive enhancer element is involved in the senescence-related expression of the carnation glutathione-S-transferase (*GST1*) gene. Proc. Natl. Acad. Sci. USA.

[84] Huo Y., Zhang B., Chen L., Zhang J., Zhang X., Zhu C. (2021). Isolation and functional characterization of the promoters of miltiradiene synthase genes, *TwTPS27a* and *TwTPS27b*, and interaction analysis with the transcription factor TwTGA1 from *Tripterygium wilfordii*. Plants.

[85] Verma V., Ravindran P., Kumar P.P. (2016). Plant hormone-mediated regulation of stress responses. BMC Plant Biol..

[86] He C., Liu X., Teixeira da Silva J.A., Wang H., Peng T., Zhang M., Si C., Yu Z., Tan J., Zhang J. (2021). Characterization of LEA genes in *Dendrobium officinale* and one gene in induction of callus. J. Plant Physiol..

[87] Tanin M.J., Saini D.K., Sandhu K.S., Pal N., Gudi S., Chaudhary J., Sharma A. (2022). Consensus genomic regions associated with multiple abiotic stress tolerance in wheat and implications for wheat breeding. Sci. Rep..

