# Supplementary material for: Genome-Wide Identification and Expression Analysis of the NAC Gene Family in Kandelia obovata, a Typical Mangrove Plant
Source: Curr Issues Mol Biol. 2022 Nov 13;44(11):5622–37. doi: 10.3390/cimb44110381 (PMC9689236; doi:10.3390/cimb44110381)
Supplement: Supplementary file 1 [file cimb-44-00381-s001.zip › Figure S1.R1.pdf]

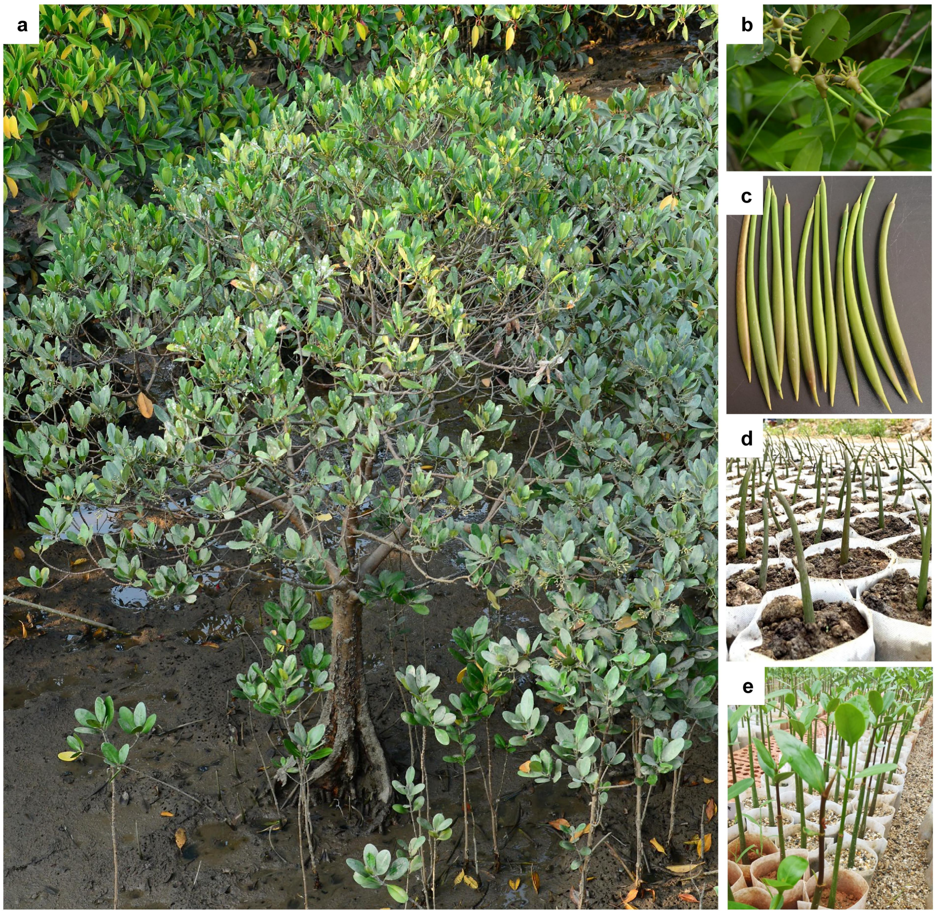

Figure S1. Morphological features of *K. obovata*. (**a**) *K. obovata* tree in the coastal wetland of Guangxi Maoweihai Mangrove Nature Reserve, Qinzhou, China. (**b**) Young fruits with viviparous trait. (**c**) The sampled healthy mature propagules. (**d**) The propagules were cultured in rooting media for 5 days. (**e**) The growing seedlings were cultured from the propagules for 3 weeks.
