# Supplementary material for: Genome-Wide Identification and Expression Analysis of the NAC Gene Family in Kandelia obovata, a Typical Mangrove Plant
Source: Curr Issues Mol Biol. 2022 Nov 13;44(11):5622–37. doi: 10.3390/cimb44110381 (PMC9689236; doi:10.3390/cimb44110381)
Supplement: Supplementary file 1 [file cimb-44-00381-s001.zip › Table S1_R1.pdf]

Table S1. The basic information of *Arabidopsis* NAC genes

Name	Gene ID	Class	Intron number	Average intron length (bp)	Protein length (aa)	pI
ANAC001	AT1G01010.1	V	5	117	430	4.89
ANAC002	AT1G01720.1	VII	2	88	290	6.53
ANAC003	AT1G02220.1	V	5	97	395	5.67
ANAC004	AT1G02230.1	V	5	105	360	6.52
ANAC005	AT1G02250.1	V	5	105	363	7.72
ANAC006	AT1G03490.1	I	3	63	282	4.35
ANAC007	AT1G12260.1	IX	3	384	396	6.23
ANAC008	AT1G25580.1	III	5	238	450	4.69
ANAC009	AT1G26870.1	VII	3	284	426	6.51
ANAC010	AT1G28470.1	III	2	333	315	7.62
ANAC011	AT1G32510.1	VIII	3	90	284	5.11
ANAC012	AT1G32770.1	IX	2	267	359	5.58
ANAC013	AT1G32870.1	VI	2	182	529	4.77
ANAC014	AT1G33060.2	V	6	171	653	5.77
ANAC015	AT1G33280.1	IX	2	89	306	8.07
ANAC016	AT1G34180.2	VI	4	167	577	4.97
ANAC017	AT1G34190.1	VI	3	172	558	4.56
ANAC018	AT1G52880.1	VII	2	93	321	8.57
ANAC019	AT1G52890.1	VII	2	101	318	6.41
ANAC020	AT1G54330.1	VIII	2	136	302	5.47
ANAC021	AT1G56010.2	X	2	660	325	7.27
ANAC022	AT1G56010.1	X	1	103	257	8.21
ANAC023	AT1G60280.1	III	0	0	348	7.28
ANAC024	AT1G60350.1	III	0	0	321	7.62
ANAC025	AT1G61110.1	VII	2	178	324	8.14
ANAC026	AT1G62700.1	IX	2	258	395	6.64
ANAC027	AT1G64105.1	V	0	0	162	5.87
ANAC028	AT1G65910.1	VIII	5	257	632	6.62
ANAC029	AT1G69490.1	VII	2	93	269	6.86
ANAC030	AT1G71930.1	IX	2	326	325	6.51
ANAC031	AT1G76420.1	X	2	402	335	6.46
ANAC032	AT1G77450.1	VII	2	97	254	8.66
ANAC033	AT1G79580.1	IX	3	339	372	7.22
ANAC034	AT2G02450.1	VII	3	490	379	6.51
ANAC035	AT2G02450.2	VII	2	682	415	6.65
ANAC036	AT2G17040.1	VII	2	85	277	9.25
ANAC037	AT2G18060.1	IX	2	405	366	5.23
ANAC038	AT2G24430.1	X	3	696	317	8.43
ANAC039	AT2G24430.2	X	2	961	316	8.43
ANAC040	AT2G27300.1	V	3	178	336	6.28

Table S1 (continued). The basic information of *Arabidopsis* NAC genes

Name	Gene ID	Class	Intron number	Average intron length (bp)	Protein length (aa)	pI
ANAC041	AT2G33480.1	VII	2	84	269	9.51
ANAC042	AT2G43000.1	VII	2	571	276	8.30
ANAC043	AT2G46770.1	IX	2	405	366	6.75
ANAC044	AT3G01600.1	III	4	158	371	5.50
ANAC045	AT3G03200.1	VIII	5	190	480	5.34
ANAC046	AT3G04060.1	X	2	157	339	6.79
ANAC047	AT3G04070.1	VII	2	163	360	7.03
ANAC048	AT3G04420.1	V	5	86	343	5.09
ANAC049	AT3G04430.1	V	3	296	199	5.84
ANAC050	AT3G10480.3	VI	7	114	527	6.34
ANAC051	AT3G10490.1	VI	4	304	238	7.74
ANAC052	AT3G10490.2	VI	4	315	452	5.42
ANAC053	AT3G10500.1	VI	5	105	550	4.37
ANAC054	AT3G15170.1	X	2	297	311	8.20
ANAC055	AT3G15500.1	VII	2	100	318	8.87
ANAC056	AT3G15510.1	VII	2	125	365	7.74
ANAC057	AT3G17730.1	VIII	2	348	247	4.67
ANAC058	AT3G18400.1	X	2	453	315	6.52
ANAC059	AT3G29035.1	X	2	107	319	8.42
ANAC060	AT3G44290.1	V	3	369	336	5.29
ANAC061	AT3G44350.2	VII	2	464	242	8.64
ANAC062	AT3G49530.1	V	5	135	470	5.94
ANAC063	AT3G55210.1	II	2	93	281	6.13
ANAC064	AT3G56530.1	II	2	89	320	5.43
ANAC065	AT3G56560.1	II	2	390	229	9.63
ANAC066	AT3G61910.1	IX	1	211	335	6.04
ANAC067	AT4G01520.1	V	3	622	303	5.57
ANAC068	AT4G01540.1	V	5	146	474	6.10
ANAC069	AT4G01550.1	V	5	166	458	5.26
ANAC070	AT4G10350.1	IX	2	260	342	7.26
ANAC071	AT4G17980.1	VIII	4	101	263	8.38
ANAC072	AT4G27410.3	VII	2	72	315	8.64
ANAC073	AT4G28500.1	III	2	169	306	9.27
ANAC074	AT4G28530.1	X	2	1411	353	6.07
ANAC075	AT4G29230.1	III	5	625	499	7.04
ANAC076	AT4G36160.1	IX	3	644	378	6.61
ANAC077	AT5G04400.1	VI	3	206	396	5.18
ANAC078	AT5G04410.1	VI	5	148	568	4.38
ANAC079	AT5G07680.1	X	2	122	330	9.18
ANAC080	AT5G07680.2	X	4	109	315	9.70

Table S1 (continued). The basic information of *Arabidopsis* NAC genes

Name	Gene ID	Class	Intron number	Average intron length (bp)	Protein length (aa)	pI
ANAC081	AT5G08790.1	VII	2	91	284	5.47
ANAC082	AT5G09330.1	VI	4	217	490	4.47
ANAC083	AT5G13180.1	VII	2	89	253	9.14
ANAC084	AT5G14000.1	IV	2	120	207	9.48
ANAC085	AT5G14490.1	III	3	81	351	6.79
ANAC086	AT5G17260.1	VIII	5	233	477	5.23
ANAC087	AT5G18270.1	X	2	249	336	6.11
ANAC088	AT5G18300.1	V	0	0	148	5.37
ANAC089	AT5G22290.1	V	3	145	341	5.21
ANAC090	AT5G22380.1	VII	2	205	236	5.77
ANAC091	AT5G24590.2	V	4	113	452	5.85
ANAC092	AT5G39610.1	X	2	102	286	5.94
ANAC093	AT5G39690.1	II	3	378	295	4.63
ANAC094	AT5G39820.1	VII	2	90	338	8.67
ANAC095	AT5G41090.1	IV	1	136	213	4.24
ANAC096	AT5G46590.1	VIII	3	86	293	4.95
ANAC097	AT5G50820.1	I	2	101	194	4.68
ANAC098	AT5G53950.1	X	2	352	376	8.52
ANAC099	AT5G56620.1	III	4	366	387	8.77
ANAC100	AT5G61430.1	X	2	109	337	8.20
ANAC101	AT5G62380.1	IX	1	129	349	5.01
ANAC102	AT5G63790.1	VII	2	95	313	8.65
ANAC103	AT5G64060.1	VI	4	148	357	4.88
ANAC104	AT5G64530.1	IV	2	389	188	4.69
ANAC105	AT5G66300.1	IX	2	183	293	6.41
