# Supplementary material for: Genome-Wide Identification and Expression Analysis of the NAC Gene Family in Kandelia obovata, a Typical Mangrove Plant
Source: Curr Issues Mol Biol. 2022 Nov 13;44(11):5622–37. doi: 10.3390/cimb44110381 (PMC9689236; doi:10.3390/cimb44110381)
Supplement: Supplementary file 1 [file cimb-44-00381-s001.zip › Table S2_R1.pdf]

Table S2. The primers of *KoNAC* genes in this study

Gene Name	Forward primer (5'-3')	Reverse primer (5'-3')
KoNAC6	TGAGGTCACGTGTGAGAAGG	CATGTCTTGCGAAGGTGAGA
KoNAC15	CGCCCAGAAATAGAAATGGA	CACTCTGGACCTCCTTCTCG
KoNAC20	CTCTGCAGCACGTGGTTTTA	GGGGTCCTGGTTCCAGTTAT
KoNAC38	GCCTGCAGAGTGGATAGGAG	GATGCATTGCTGTCTCTGGA
KoNAC51	TTGCGGTCTACCTGCTCTTT	ATCCAGTCCTCGTCCCTTTT
KoActin	CAATGCAGCAGTTGAAGGAA	CTGCTGGAAGGAACCAAGAG
