# Supplementary material for: Genome-Wide Identification and Expression Analysis of the NAC Gene Family in Kandelia obovata, a Typical Mangrove Plant
Source: Curr Issues Mol Biol. 2022 Nov 13;44(11):5622–37. doi: 10.3390/cimb44110381 (PMC9689236; doi:10.3390/cimb44110381)
Supplement: Supplementary file 1 [file cimb-44-00381-s001.zip › Table S3_R1.pdf]

Table S3. Common orthologous gene pairs of *K. obovata* between *A. thaliana* and *P. trichocarpa*

Number	K. obovata	Arabidopsis	P. trichocarpa
1	KoNAC1	AT3G04060.1	POPTR_0017s12210.1
2	KoNAC10	AT5G07680.1	POPTR_0001s35490.1
3	KoNAC12	AT3G10480.3	POPTR_0013s05080.1
4	KoNAC14	AT4G29230.1	POPTR_0013s11740.1
5	KoNAC18	AT5G39820.1	POPTR_0002s08150.1
6	KoNAC19	AT3G01600.1	POPTR_0014s04080.1
7	KoNAC2	AT5G18270.1	POPTR_0017s11830.1
8	KoNAC20	AT2G17040.1	POPTR_0002s00730.1
9	KoNAC23	AT3G04070.1	POPTR_0002s06210.1
10	KoNAC24	AT1G71930.1	POPTR_0001s41490.1
11	KoNAC25	AT1G34180.2	POPTR_0001s41460.1
12	KoNAC26	AT1G77450.1	POPTR_0001s40680.1
13	KoNAC28	AT1G76420.1	POPTR_0014s10330.1
14	KoNAC29	AT1G34180.2	POPTR_0014s10320.1
15	KoNAC31	AT1G52880.1	POPTR_0014s10060.1
16	KoNAC32	AT4G27410.3	POPTR_0002s05820.1
17	KoNAC33	AT3G15170.1	POPTR_0001s13380.1
18	KoNAC34	AT4G28530.1	POPTR_0017s12210.1
19	KoNAC35	AT4G01540.1	POPTR_0012s03100.1
20	KoNAC36	AT4G01520.1	POPTR_0012s05280.1
21	KoNAC37	AT2G46770.1	POPTR_0018s06790.1
22	KoNAC38	AT2G43000.1	POPTR_0010s23630.1
23	KoNAC39	AT5G50820.1	POPTR_0018s03910.1
24	KoNAC4	AT2G18060.1	POPTR_0017s12210.1
25	KoNAC41	AT5G07680.1	POPTR_0001s02710.1
26	KoNAC43	AT3G18400.1	POPTR_0001s45250.1
27	KoNAC44	AT4G29230.1	POPTR_0014s10320.1
28	KoNAC45	AT3G10480.3	POPTR_0002s03830.1
29	KoNAC46	AT2G24430.1	POPTR_0011s05740.1
30	KoNAC47	AT4G17980.1	POPTR_0001s13380.1

Table S3 (continued). Common orthologous gene pairs of *K. obovata* between *Arabidopsis* and poplar

Number	K. obovata	Arabidopsis	P. trichocarpa
31	KoNAC48	AT5G62380.1	POPTR_0010s17350.1
32	KoNAC49	AT2G46770.1	POPTR_0010s13980.1
33	KoNAC5	AT5G09330.1	POPTR_0001s33260.1
34	KoNAC51	AT4G01520.1	POPTR_0001s13380.1
35	KoNAC54	AT4G28530.1	POPTR_0001s45700.1
36	KoNAC55	AT4G28500.1	POPTR_0001s00220.1
37	KoNAC56	AT5G13180.1	POPTR_0001s02710.1
38	KoNAC57	AT5G62380.1	POPTR_0001s22630.1
39	KoNAC58	AT1G25580.1	POPTR_0001s41460.1
40	KoNAC59	AT5G13180.1	POPTR_0001s02710.1
41	KoNAC6	AT5G63790.1	POPTR_0008s03170.1
42	KoNAC60	AT5G24590.2	POPTR_0001s45700.1
43	KoNAC61	AT5G62380.1	POPTR_0010s17350.1
44	KoNAC62	AT4G17980.1	POPTR_0001s13380.1
45	KoNAC64	AT3G44350.2	POPTR_0001s22630.1
46	KoNAC66	AT3G15500.1	POPTR_0002s05820.1
47	KoNAC7	AT3G29035.1	POPTR_0018s06790.1
48	KoNAC8	AT5G39820.1	POPTR_0007s04780.1
49	KoNAC9	AT5G50820.1	POPTR_0017s11830.1
