# Supplementary material for: Genome-Wide Identification and Expression Analysis of the NAC Gene Family in Kandelia obovata, a Typical Mangrove Plant
Source: Curr Issues Mol Biol. 2022 Nov 13;44(11):5622–37. doi: 10.3390/cimb44110381 (PMC9689236; doi:10.3390/cimb44110381)
Supplement: Supplementary file 1 [file cimb-44-00381-s001.zip › Table S4_R1.pdf]

Table S4. The FPKM values of *KoNAC* genes expressed in different organs

Genes	root	stem	leaf	flower	sepal	stamen	pistil	fruit
KoNAC1	3.13	17.80	18.75	25.46	21.44	21.01	13.25	42.47
KoNAC2	5.90	3.06	4.21	1.33	4.99	0.45	3.11	1.39
KoNAC3	0.18	0.00	0.46	0.85	0.16	0.18	0.87	0.24
KoNAC4	21.38	7.41	8.38	2.89	2.27	2.50	6.44	8.34
KoNAC5	51.51	72.15	70.58	47.65	41.13	50.08	48.37	30.77
KoNAC6	64.95	26.92	35.32	327.95	29.17	299.22	32.58	390.28
KoNAC7	5.60	0.66	0.94	12.36	1.19	13.91	10.87	16.33
KoNAC8	1.90	0.29	0.35	0.13	0.16	0.21	0.04	0.18
KoNAC9	1.03	3.45	1.74	0.27	0.35	0.45	0.19	3.15
KoNAC10	4.40	1.95	1.48	7.32	16.43	1.73	5.91	1.43
KoNAC11	13.49	24.43	21.73	4.47	1.18	2.38	5.65	10.07
KoNAC12	56.23	69.62	67.25	55.50	62.30	61.16	60.64	51.43
KoNAC13	28.55	24.71	17.45	14.77	15.65	13.30	12.23	15.45
KoNAC14	6.74	9.42	7.99	3.65	2.22	3.25	3.73	2.65
KoNAC15	30.73	4.12	5.35	69.78	39.15	110.86	30.17	189.58
KoNAC16	1.87	0.60	0.80	1.47	1.02	1.70	1.90	1.12
KoNAC17	0.00	0.46	0.67	1.13	0.25	0.98	2.59	0.21
KoNAC18	5.66	0.21	8.89	1.24	4.31	0.31	0.45	1.10
KoNAC19	3.58	4.39	2.85	0.17	1.20	0.27	0.69	1.18
KoNAC20	200.97	107.82	73.71	16.12	6.96	3.39	2.88	48.37
KoNAC21	0.92	0.23	1.35	0.19	0.14	0.43	0.31	0.31
KoNAC22	0.00	0.00	5.79	0.08	0.88	0.00	0.00	0.00
KoNAC23	7.26	2.13	13.14	18.13	10.49	16.98	6.05	9.16
KoNAC24	0.37	0.63	0.83	0.21	0.39	0.22	0.33	4.51
KoNAC25	33.88	40.55	51.26	22.88	22.44	40.30	21.53	25.41
KoNAC26	126.75	47.51	47.03	515.94	197.70	524.54	79.06	658.36
KoNAC27	39.06	48.13	38.58	33.76	18.79	66.19	24.30	56.15
KoNAC28	0.68	0.48	0.15	1.07	0.81	1.74	4.63	0.02
KoNAC29	120.48	81.62	78.11	46.96	41.48	47.11	30.02	58.43
KoNAC30	2.30	0.30	2.14	0.92	0.19	0.00	0.00	0.11
KoNAC31	4.38	7.38	3.83	1.73	5.52	0.61	0.18	36.32
KoNAC32	11.60	2.42	4.03	53.83	33.21	115.56	13.58	83.33
KoNAC33	0.00	0.00	0.21	0.15	0.08	0.62	0.20	0.11
KoNAC34	1.29	0.00	0.21	0.00	0.00	0.71	0.00	0.05
KoNAC35	11.50	4.93	0.81	4.04	2.42	11.87	3.23	9.31
KoNAC36	0.31	0.37	0.46	0.40	0.00	0.00	0.00	0.37
KoNAC37	11.89	10.89	0.42	14.95	0.00	37.32	0.00	1.04
KoNAC38	5.81	2.83	4.27	1.02	0.21	0.59	3.45	5.38
KoNAC39	2.86	1.86	3.29	1.97	2.26	1.84	0.99	5.57

Table S4 (continued). The FPKM values of *KoNAC* genes expressed in different organs

Genes	root	stem	leaf	flower	sepal	stamen	pistil	fruit
KoNAC41	14.41	0.40	3.57	12.16	21.78	24.74	5.00	3.74
KoNAC42	5.24	2.23	0.44	0.53	0.36	0.52	0.70	2.12
KoNAC43	3.74	0.51	0.37	0.00	0.00	0.00	0.00	2.27
KoNAC44	6.99	10.55	9.09	4.09	3.17	3.07	6.67	4.10
KoNAC45	26.79	29.53	25.18	25.96	23.60	19.70	23.14	12.01
KoNAC46	29.44	0.27	0.79	0.53	0.00	0.05	0.00	0.94
KoNAC47	4.32	3.88	3.31	3.41	1.07	2.08	3.62	1.38
KoNAC48	1.71	2.36	0.73	0.58	0.00	0.33	0.36	0.84
KoNAC49	4.12	14.49	0.60	0.48	0.78	0.35	1.29	0.16
KoNAC50	2.59	2.10	21.20	78.09	32.17	99.81	1.99	198.42
KoNAC51	19.85	12.71	12.66	24.12	18.86	156.64	18.17	27.21
KoNAC52	31.32	18.43	28.21	8.68	8.59	8.70	5.21	5.13
KoNAC53	0.13	0.04	0.78	1.00	0.04	1.45	2.64	0.70
KoNAC54	69.33	7.01	3.70	3.29	0.59	2.57	4.51	6.90
KoNAC55	5.17	10.34	1.68	16.76	2.58	37.82	1.06	2.33
KoNAC56	53.34	30.93	132.40	30.27	40.57	27.56	35.75	34.66
KoNAC57	10.13	10.54	19.99	9.82	3.58	7.43	0.00	20.42
KoNAC58	16.91	25.32	7.61	17.44	13.51	20.06	24.42	18.85
KoNAC59	46.73	52.72	101.96	44.08	89.48	36.83	27.20	25.95
KoNAC60	165.07	70.98	84.14	61.77	68.21	60.43	78.37	65.88
KoNAC61	2.22	2.69	1.17	0.00	0.00	0.00	0.61	0.17
KoNAC62	6.19	9.65	2.22	2.17	1.94	3.40	5.73	2.02
KoNAC63	28.44	13.63	3.46	12.01	1.80	19.27	0.08	3.11
KoNAC64	5.09	0.26	0.33	0.00	0.00	0.28	0.17	1.31
KoNAC65	0.38	0.89	1.34	0.00	0.22	0.04	0.00	0.32
KoNAC66	0.00	0.00	0.14	0.33	0.08	0.17	0.00	0.00
KoNAC68	11.00	10.57	2.47	3.24	2.04	15.35	2.00	4.72
