# Supplementary material for: Genome-Wide Identification and Expression Analysis of the NAC Gene Family in Kandelia obovata, a Typical Mangrove Plant
Source: Curr Issues Mol Biol. 2022 Nov 13;44(11):5622–37. doi: 10.3390/cimb44110381 (PMC9689236; doi:10.3390/cimb44110381)
Supplement: Supplementary file 1 [file cimb-44-00381-s001.zip › Table S5_R1.pdf]

Table S5. The FPKM values of differentially expressed *KoNAC* genes under cold treatment

Genes	CK	cold 1	cold 2	cold 4
KoNAC6	20.86	15.44	667.83	812.82
KoNAC11	31.02	26.41	30.03	79.13
KoNAC15	2.32	4.75	152.24	264.52
KoNAC20	57.62	29.77	498.41	494.05
KoNAC24	0.10	0.22	0.53	2.74
KoNAC26	278.28	74.47	2149.77	2253.79
KoNAC32	2.02	0.84	10.02	50.99
KoNAC35	2.18	0.85	15.71	11.29
KoNAC38	5.82	2.28	33.67	212.60
KoNAC41	2.09	2.35	6.54	11.82
KoNAC51	24.63	5.40	26.59	12.65
KoNAC62	0.47	1.36	1.61	6.67
KoNAC68	3.24	1.99	6.54	29.21

Cold 1, first-time cold treatment; Cold 2, second-time cold treatment; Cold 4, fourth-time cold treatment.
