# Supplementary material for: Genome-Wide Identification and Expression Analysis of the NAC Gene Family in Kandelia obovata, a Typical Mangrove Plant
Source: Curr Issues Mol Biol. 2022 Nov 13;44(11):5622–37. doi: 10.3390/cimb44110381 (PMC9689236; doi:10.3390/cimb44110381)
Supplement: Supplementary file 1 [file cimb-44-00381-s001.zip › Table S6_R1.pdf]

Table S6. Number of stress-related cis-regulatory elements in the promoter regions of the differentially expressed *KoNAC* genes.

Genes	LTR	STRE	ARE	ABRE	ERE	TGACG-motif	WRE3	WUN
KoNAC6	0	1	1	7	1	4	4	0
KoNAC11	1	1	1	2	0	0	1	0
KoNAC15	0	1	1	6	1	12	0	1
KoNAC20	1	5	2	1	1	1	1	1
KoNAC24	1	1	0	2	0	6	2	1
KoNAC26	0	2	0	9	2	8	0	0
KoNAC32	0	1	0	15	1	4	0	0
KoNAC35	0	3	0	5	1	6	0	2
KoNAC38	2	4	3	4	0	4	1	0
KoNAC41	1	2	4	5	2	0	0	1
KoNAC51	1	0	2	3	1	4	0	2
KoNAC62	2	1	0	3	0	2	0	0
KoNAC68	1	1	2	4	1	0	1	1
